# Variation in responses to incretin therapy: Modifiable and non-modifiable factors

**DOI:** 10.3389/fmolb.2023.1170181

**Published:** 2023-04-07

**Authors:** Gregory O. Austin, Alejandra Tomas

**Affiliations:** Section of Cell Biology and Functional Genomics, Division of Diabetes, Endocrinology and Metabolism, Department of Metabolism, Digestion and Reproduction, Imperial College London, London, United Kingdom

**Keywords:** incretin—based therapy, GLP-1 (glucagon-like peptide-1), GIP (glucose-dependent insulinotropic polypeptide), T2D (type 2 diabetes), obesity, incretin receptors

## Abstract

Type 2 diabetes (T2D) and obesity have reached epidemic proportions. Incretin therapy is the second line of treatment for T2D, improving both blood glucose regulation and weight loss. Glucagon-like peptide-1 (GLP-1) and glucose-stimulated insulinotropic polypeptide (GIP) are the incretin hormones that provide the foundations for these drugs. While these therapies have been highly effective for some, the results are variable. Incretin therapies target the class B G protein-coupled receptors GLP-1R and GIPR, expressed mainly in the pancreas and the hypothalamus, while some therapeutical approaches include additional targeting of the related glucagon receptor (GCGR) in the liver. The proper functioning of these receptors is crucial for incretin therapy success and here we review several mechanisms at the cellular and molecular level that influence an individual’s response to incretin therapy.

## Introduction

Type 2 diabetes (T2D) is one of the major health concerns affecting ∼9% of the global population, putting those affected at risk of cardiovascular-associated mortality (Editors, 2021). T2D is characterized by elevated blood glucose levels resulting from a combined failure of pancreatic beta cell function (reduced or dysregulated insulin secretion) and glucose clearance by peripheral tissues (insulin resistance) (James et al., 2021).

The incretins glucagon-like peptide-1 (GLP-1) and glucose-stimulated insulinotropic polypeptide (GIP) are peptide hormones secreted from intestinal enteroendocrine cells (Baggio and Drucker, 2007) that regulate blood glucose levels by potentiating insulin secretion in response to nutrient intake as well as promoting beta cell survival (Nauck and Meier, 2018), with the “incretin effect” referring to the greater amount of insulin secreted in response to an oral glucose load relative to an equal intravenous dose. Incretin mimetics are ideal candidate therapies for T2D as they exert their actions specifically during hyperglycaemic conditions and have the additional capacity to regulate body weight (Kim and Egan, 2008).

Since 2005, six incretin therapy drugs have been approved by the FDA (United States) and the MHRA (United Kingdom) for T2D treatment: Exenatide, Lixisenatide, Liraglutide, Dulaglutide, Semaglutide and Tirzepatide, all of which are administered daily or once weekly *via* subcutaneous injection; although Semaglutide is now also available as an oral medication (Latif et al., 2022). Initially, incretin therapies focused on synthesizing GLP-1 analogues that were resistant to degradation by dipeptidyl peptidase-4 (DPP-4), which rapidly breaks down endogenous GLP-1 (Röhrborn et al., 2015). Exenatide, purified from the Gila monster *Heloderma suspectum*, has an increased binding affinity to the GLP-1 receptor (GLP-1R) and reduced rate of degradation by DPP-4 compared to GLP-1, increasing its half-life from 1 min to over 2 h (Gao and Jusko, 2011).

Three classes of DPP-4 inhibitors (Gliptins) are in clinical use to augment the endogenous incretin effect, and these provide 80%–90% efficacy in inhibiting the peptidase; however, DPP-4 inhibitors are not administered in conjunction with incretin therapies (Gallwitz, 2019).

All GLP-1R agonists reduce food intake, body weight, and HbA1c and improve both blood pressure and blood lipid profiles. These benefits were highlighted in 2019 when the Lancet published a meta-analysis of seven clinical trials showing that these treatments reduced hospital admission by 9%, all-cause mortality by 12% and deaths associated with kidney disease by 17% (Kristensen et al., 2019). Then, in 2021, Semaglutide administered once weekly over 68 weeks was shown to result in 32% of participants losing >20% of their body mass compared to only 1.7% in controls. This study also showed that 86.4% of individuals lost ≥ 5% of their body mass (Wilding et al., 2021). Moreover, last year a separate study showed that 91% of obese individuals receiving a weekly dose of 15 mg Tirzepatide—a recently developed GLP-1R/GIPR dual agonist, lost 5% of their body mass with 36% of participants on the same dosage losing at least 25% (Jastreboff et al., 2022).

While these data are astounding—with yearly progress in efficacy, we should consider that around 9% of patients receiving these game-changer treatments (Killworth, 2021) were not able to lose even 5% of their body mass over 72 weeks, and some trials are still struggling to get participants’ HbA1c below 7% (Table 1). Additionally, incretin therapies can have overbearing negative side effects including hypoglycaemia (often in combination with other glucose-lowering treatments), nausea, vomiting, gastrointestinal upset (including diarrhoea) and injection site reactions that contribute to therapy discontinuation (Trujillo et al., 2021); this review highlights the current research investigating the modifiable and non-modifiable factors contributing to the ‘non-responder’ phenotype to incretin therapies.

**TABLE 1:** Incretin therapy studies including percentage of participants who failed to reach the desired endpoint.

Drug	Trial name	Year	(n)	Dose and frequency	Duration (weeks)	Target endpoint	Failure to reach endpoint (%)	Drop out due to AE (%)	Contributing factors	References
Exenatide		2005	336	10 ug twice daily	30	HbA1c <7.0%	54	7.1	Metformin	Defronzo et al. (2005)
	LEAD-6	2009	231	10 ug twice daily	26	HbA1c <7.0%	57	13.4	Metformin and Sulfonylurea	Buse et al. (2009)
		2015	100	5 ug twice daily for 4 weeks then 10 ug twice daily	26	5% loss of body mass	13	19	Metformin and Sulfonylurea, all awaiting bariatric surgery	Iglesias et al. (2015)
Liraglutide	vs. Lixisenatide	2016	202	1.8 mg daily	26	HbA1c <7.0% and no weight gain	33.5	6.4	Metformin	Nauck et al. (2016)
	LEAD-6	2009	233	1.8 mg daily	26	HbA1c <7.0%	46	10.3	Metformin and Sulfonylurea	Buse et al. (2009)
Lixisenatide	GetGoal-Mono	2012	119	10–20 ug (1 step) daily	12	HbA1c <7.0%	53.5	2.5		Fonseca et al. (2012)
	GetGoal-Mono	2012	120	10–20 ug (2 step) daily	12	HbA1c <7.0%	47.8	4.2		Fonseca et al. (2012)
	vs. Liraglutide	2016	202	20 ug daily	26	HbA1c <7.0% and no weight gain	58.1	7.4	Metformin	Nauck et al. (2016)
Dulaglutide	AWARD -1	2014	279	1.5 mg Weekly	26	HbA1c < 7.0%	22	3.2	Metformin and Glitazone	Wysham et al. (2014)
	AWARD-2	2015	273	1.5 mg Weekly	78	HbA1c < 7.0%	46.8	3.3	Metformin and Sulfonylurea	Giorgino et al. (2015)
Semaglutide	STEP 1	2021	1306	2.4 mg once weekly	68	5% loss of body mass	13.6	7.0	Adults, includes lifestyle intervention	Wilding et al. (2021)
	STEP TEEN	2022	134	2.4 mg once weekly	68	5% loss of body mass	27	5.0	Adolescents (<18 years), includes lifestyle intervention	Weghuber et al. (2022)
Tirzepatide	SURMOUNT-1	2022	630	15 mg once weekly	72	5% loss of body mass	9	6.2	Majority Caucasian decent (70.6%)	Jastreboff et al. (2022)

## Incretin signalling and biased agonism: Basic concepts

Both GLP-1 and GIP stimulate insulin secretion from pancreatic beta cells, with GIP also stimulating alpha cells to secrete glucagon (Manchanda et al., 2022). These functions occur *via* binding and activation of their cognate secretin-like (class B) G protein-coupled receptors (GPCRs). Orthosteric binding of incretin peptides to the N-terminus (extracellular) region of the receptors causes a conformational change, stabilizing a binding site in the intracellular region that allows coupling to and activation of heterotrimeric guanine nucleotide-binding protein (G proteins) (Chen and Tesmer, 2022). Heterotrimeric G protein activation causes their *a* and *βγ* subunits to dissociate triggering different signalling cascades. For the GLP-1R and the GIPR [and also relevant for the other member of the family, the glucagon receptor (GCGR)], the predominant G protein recruited is Gα_s_ that activates adenylate cyclase (mainly isoforms 5 and 8), triggering the production of the second messenger cyclic adenosine monophosphate (cAMP) from intracellular ATP (Anton et al., 2022) and subsequent downstream signalling. Alternatively, Gα_i_ subunits inhibit adenylate cyclase activity and Gα_q_ triggers the mobilisation of Ca^2+^ ions *via* phospholipase C_β_ activity that produces inositol-1,4,5 triphosphate (IP_3_) from phosphatidylinositol 4-5-bisphosphate (PIP_2_). Additionally, dissociated Gβγ subunits can also trigger signalling that leads to activation of both the calcium and the extracellular signal-regulated kinase (ERK1/2) pathways (Lymperopoulos et al., 2022).

After activation of signal transduction, the process is then turned off in a stepwise manner. G protein-coupled receptor kinases (GRKs) phosphorylate specific sites at the receptor C-terminal tail, providing binding sites for β-arrestins (Arcones et al., 2021), scaffold proteins involved in the desensitization and internalization of GPCRs, as well as engaging in their specific signalling (van Gastel et al., 2018). When coupled to β-arrestins, GPCRs are desensitized by abrogated coupling to G proteins and trafficked away from the cell surface, destined for either re-sensitization following their recycling back to the plasma membrane, or for final signal termination resulting from their degradation in lysosomes (McCorvy et al., 2017). Additionally, internalized receptors and GPCR:β-arrestin complexes can continue to signal from the endosome, with this process potentially engaging alternative signalling cascades (Caengprasath et al., 2020). In this context, ligand-associated biased agonism is defined as the observation that certain ligands (in comparison to a reference compound) specifically favour either G protein or β-arrestin transducer pathways—within a particular GPCR (Jones, 2021; Kolb et al., 2022), an effect that has been described for the GLP-1R and exploited by some GLP-1R agonists in clinical use such as Tirzepatide (Willard et al., 2020). While beneficial effects are observed when compounds trigger bias at the GLP-1R towards G protein signalling (Jones et al., 2018), complete blockade of β-arrestin action is detrimental (Kuna et al., 2013). This beneficial effect is evident in the G protein-biased agonist Exendin-phe1, derived from a single phenylalanine substitution at position 1 in Exenatide, which displays improved receptor recycling, reduced receptor desensitization, and sustained incretin-stimulated insulin secretion (Jones et al., 2018). Similar G protein-biased agonists have also been developed for the GIPR and the GCGR, but the full extent of their effects on beta cell function is still unknown (Jones et al., 2021).

## Genetic variation at the incretin receptors

The most direct answer to why people fail to respond to incretin therapies is that they might express non-functional incretin receptors. Genetic missense variants in GLP-1R, GIPR and GCGR have been identified, with both gain of function (GoF) and loss of function (LoF) effects. GoF receptors are often characterised by a greater propensity for G protein versus β-arrestin recruitment and signalling, slower rate of receptor internalisation and rapid recycling back to the plasma membrane, with these characteristics being independent of their surface expression levels (Liu et al., 2022). LoF variants may be misfolded, reducing successful biosynthesis and surface expression; or they might display enhanced β-arrestin recruitment, higher degradation rates and/or defective ligand binding ability (Tao and Conn, 2014). A missense variant in the GIPR, E354Q—whereby a glutamine (Q) at amino acid position 354 replaces a glutamic acid (E), identified in a genome-wide association study (GWAS), is linked with increased risk of T2D and higher BMI (Speliotes et al., 2010). GIPR E354Q was characterized in differentiated adipocytes as having a higher rate of desensitization and reduced recycling capacity, all markers of enhanced β-arrestin recruitment. While these features are associated with a LoF phenotype, other studies suggest that both GIPR agonists and antagonists may provide benefits to T2D and obesity (Yang et al., 2022), indicating a complex mechanism of GIPR action within the processes of blood glucose and weight regulation. A suggested mechanism involves prolonged GIPR agonism causing receptor degradation, reducing sustained GIP signalling and, when combined with GLP-1R agonism, causing increased weight loss (Killion et al., 2020). However, it remains an open question how both activation and inhibition of GIPR can lead to beneficial effects, and whether the presence of the E354Q variant at the GIPR may affect the efficacy of GLP-1R agonists (GLP-1RAs).

The GLP-1R has itself several LoF variants including R380C, shown by two independent groups to have normal surface expression but reduced affinity for Exenatide (Wootten et al., 2016; Hegron et al., 2023). Another LoF variant, R421W, displays reduced coupling to mini-Gα_s_ proteins in response to various endogenous and clinical agonists (Lagou et al., 2021). Additionally, the T149M variant is associated with increased T2D risk and has normal surface expression but reduced binding affinity for GLP-1 and Exenatide, as well as reduced cAMP production (Beinborn et al., 2005), although a study was able to rescue both cAMP and ERK1/2 signalling for this variant using the allosteric modulator Compound 2 (Koole et al., 2011). On the other hand, the GLP-1R GoF variant R131Q is associated with decreased T2D risk in the Japanese population (Suzuki et al., 2019). Patients heterozygous for R131Q secrete at least double the amount of insulin in response to GLP-1 during a hyperglycaemic clamp (Sathananthan et al., 2010). Another GoF variant, A316T, shows a 2-fold increase in cAMP accumulation (Hegron et al., 2023), and increased Ca^2+^ mobilization (which is essential for insulin secretion). Furthermore, A316T shows enhanced recruitment of Gα_s_ and endocytosis in response to oxyntomodulin, GLP-1, Semaglutide and Tirzepatide, but not Exenatide (Lagou et al., 2021). Ongoing research efforts into this variant aim to uncover the molecular factors associated with its benefits, focusing on the analysis of transducer factor recruitment and receptor trafficking patterns in response to a range of clinical and non-clinical GLP-1RAs.

## Membrane composition and incretin receptor action

Dietary polyunsaturated fatty acids are essential to human health, they play important roles in both membrane phospholipid composition, which increases plasma membrane fluidity, and provide the backbone to several signalling lipids (Baccouch et al., 2023). The ability of receptors to move within the cellular plasma membrane and into intracellular compartments is vital to regulate their function (Fang et al., 2020) and an individual’s cellular membrane composition may be altered because of diet, medications and genetic factors (Sabapathy et al., 2022). For example, mice fed a high-fat diet for 21 days have both lipid and protein compositional changes in cellular plasma membranes that result in an impaired capacity for oxidative phosphorylation, increased reactive oxygen species (ROS) production, hepatosteatosis and insulin resistance (Kahle et al., 2015). In humans, dietary related alterations in hepatocyte membrane composition, including an increase in phosphatidylethanolamines, have been found to contribute to metabolic-associated fatty liver disease through mitochondrial dysfunction causing the production of ROS, lipid accumulation and loss of insulin sensitivity (Shama et al., 2023). Additionally, in obese humans, visceral fat deposits accumulate where the membrane composition regulating protein ATB8B1 (or flippase) is upregulated (Motahari-Rad et al., 2022). These studies all indicate that diet can influence the make-up of cell membranes and thus, we hypothesise that it may have a potential direct regulatory impact on incretin receptor function in target cells.

Cholesterol is a particularly important lipid for the regulation of incretin receptor function. Lipid rafts (or cholesterol-rich nanodomains) are regions within the plasma membrane where weak binding between cholesterol and other phospholipids allows specific protein interactions to occur (Ho et al., 2022). The GLP-1R forms clusters within lipid rafts when activated with Exenatide, an effect not observed for the GIPR, which presents with a higher level of pre-clustering in basal conditions (Buenaventura et al., 2019). Palmitoylation of the GLP-1R, referring to the reversible attachment of a palmitate moiety to a cysteine residue in the C-terminal tail of the receptor, also contributes to its stabilisation within lipid rafts. Clustering is also important for the recurring activation of GLP-1R, and this capacity appears to be regulated by GLP-1R localization to these cholesterol-rich membrane nanodomains (Buenaventura et al., 2019). Although cholesterol is essential for lipid raft formation, the effects of cholesterol on GPCR function are complex (Kiriakidi et al., 2019). Results from a study where methyl-β-cyclodextrin was used to deplete cellular cholesterol showed that this lipid is required for serotonin receptor stability and preservation of signal potential (Saxena and Chattopadhyay, 2012), as well as for effective serotonin control of food intake and prevention of hyperphagia (van Galen et al., 2021). For the GCGR, however, cholesterol levels are negatively associated with receptor function as demonstrated by the fact that treatment with the cholesterol-lowering drug simvastatin increases *ex vivo* cAMP levels in response to GCG stimulation in murine hepatocytes (McGlone et al., 2022). Following these results for the GCGR, we are currently investigating the effects of statin treatment on GLP-1R function in the pancreas *in vivo* and *ex vivo*. Our preliminary findings suggest that prescribed statins may indeed be a factor that can regulate responses to GLP-1RAs. Therefore, exposure to these cholesterol-lowering drugs may impact the effectiveness of incretin-based therapies.

Receptor trafficking involves the internalisation of the active receptor: Ligand complex towards endocytic compartments, followed by its deactivation/dephosphorylation and recycling back to the cell membrane, leading to its re-sensitisation, or trafficking to lysosomal organelles for its degradation and final signal termination (McGlone et al., 2021). Additional receptor destinations involve retrograde transport towards the Golgi apparatus (Bonifacino and Rojas, 2006) or receptor incorporation into exosomes intended to be secreted, potentially playing a role in inter-cellular communication (Isola and Chen, 2016).

Evidence from primary mouse islets and clonal beta cells (INS-1 832/3) shows that Exenatide causes a greater amount of GLP-1R degradation compared to endogenous GLP-1, with this effect apparently due to differences in the susceptibility of the agonists for degradation by the endopeptidase endothelin converting enzyme 1 (ECE1) (Fang et al., 2020). Also, Huntingtin-interacting protein-1 (HIP1), a protein essential to the regulation of clathrin-mediated endocytosis (Metzler et al., 2001) is associated with the control of GLP-1R function. Data from our laboratory (Buenaventura et al., 2018) shows that HIP1 knockdown reduces G protein recruitment to the GLP-1R, with HIP1 being required for effective incretin-stimulated insulin secretion from murine and human beta cell lines and primary human islets. Additionally, in this study we also showed that the endosomal sorting nexin proteins, SNX1 and SNX27, regulate GLP-1R endosomal trafficking, with SNX1 decreasing receptor recycling to the cell membrane while SNX27 having the opposite effect. Therefore, changes in the expression levels of these endocytic trafficking regulators, or mutations in their genes resulting in changes in their function could additionally impact the capacity of GLP-1RAs to exert beneficial effects in some patients.

## Effect of the microbiome

The gut microbiome consists of trillions of microorganisms that contribute to the host digestive system, generating metabolites of which the short-chain fatty acids (SCFAs): acetate, propionate and butyrate, are primary components, produced through the fermentation of indigestible polysaccharides (O’Donnell et al., 2023). Short-chain fatty acids are, among other things, ligands for the rhodopsin-like (Class A) GPCRs free fatty acid receptors (FFARs), expressed in the gut and the endocrine pancreas. FFA2 is activated predominantly by propionate and FFA3 by butyrate [EC_50_ for acetate, propionate and butyrate at FFA2 are 431, 290 and 371 μM respectively, and at FFA3 393, 41 and 33 μM (Brown et al., 2003)].

The gut microbiome is altered in obesity and T2D, an effect known as dysbiosis, where the ratio of the main bacterial phyla (*Firmicutes* and *Bacteroidetes*) becomes imbalanced, reducing diversity while the relative abundance of certain bacteria, including opportunistic pathogens, increases (Larsen et al., 2010). A pilot study investigating gut microbiomes of 52 patients receiving either Dulaglutide or Liraglutide determined a microbial signature of GLP-1RA responders (*n* = 34) and non-responders (*n* = 18) (Tsai et al., 2022). After adjusting for baseline HbA1c and serum C-peptide, the bacteria *Bacteroides dorei* and *Lachnoclostridium sp*, both of which are associated with anti-inflammatory immunomodulation and improved gut barrier integrity (Yoshida et al., 2018), were positively associated with responders. Alternatively, *Mitsuokella multacida*, a bacterium previously shown to produce trimethylamine oxide, which contributes to atherosclerosis formation (Fu et al., 2020), correlated with GLP-1RA non-responders.

SCFAs are known to indirectly influence the incretin effect (Arora et al., 2021) through the activation of FFA2 and FFA3 on both L and K enteroendocrine cells, triggering the secretion of GLP-1 and GIP, respectively (Ørgaard et al., 2019). Both FFA2 and FFA3 are expressed in pancreatic islets (DiGruccio et al., 2016), and their direct effect on insulin responses has so far provided mixed results. *Ex vivo* analysis of both murine and human islets has linked acetate and propionate to both increased and decreased glucose-stimulated insulin secretion (GSIS) (Priyadarshini et al., 2015; Tang et al., 2015; Pingitore et al., 2017). Butyrate administered in pharmacological doses (5 mM) directly to *ex vivo* rat islets can increase GSIS (Wang et al., 2022), but this effect might potentially be due to mechanisms unrelated to its binding to FFARs, such as on chromatin remodelling (Bridgeman et al., 2021). Furthermore (Priyadarshini et al., 2015), found that primary isolated wild-type mouse islets secrete 50% more insulin in a GSIS assay following co-stimulation with acetate and Exendin-4 compared to FFA2 KO islets. In an FFA3 study, propionate signalling *via* Gα_i_ negatively impacted GLP-1R function, significantly reducing Exendin-4-stimulated potentiation of insulin secretion (Priyadarshini and Layden, 2015). This study also showed that a global KO of FFA3 results in the downregulation of GLP-1R levels in mouse islets. The authors suggest that FFA3 might act to prevent hyperphagia-induced over-secretion of insulin that would cause beta cell exhaustion during dietary-related stress (Priyadarshini et al., 2020). While some progress is being made linking the gut microbiome with obesity and T2D (Perry et al., 2016; Sanna et al., 2019; Utzschneider et al., 2020), a major question remains: Do SCFAs produced in the gut reach the pancreas in relevant concentrations to influence local receptors? Most butyrate synthesized in the gut is utilized by colonocytes, with only 1–12 µM reaching systemic circulation (Stevens and Hume, 1998). Propionate is entirely metabolised by hepatocytes (Guarner and Malagelada, 2003), and acetate, a major substrate for lipogenesis, reaches a systemic concentration of 19–160 µM (Bloemen et al., 2009), meaning that only a small fraction of the acetate produced by the microbiome would be available to reach the pancreatic islets. So, while the gut microbiome is topical, microbial metabolites might have a challenging time directly affecting incretin responses at the pancreas. Alternatively, the pancreas may host its own microbiome, synthesizing SCFAs that might directly activate cognate receptors located in pancreatic islet endocrine cells. In this context, del Castillo et al. (2019) have compared the similarities between rRNA sequences isolated from the pancreas and duodenum of 50 pancreatic cancer and 34 non-cancer patients. Their findings show that the microbial signatures of these two tissues do not differ significantly, and both contain highly diverse microbial communities. They identified that cancer patients have significantly less *Lactobacillus* in their pancreas. *Lactobacilli* are lactic acid-producing bacteria capable of generating both acetate and butyrate (Hati et al., 2019). Future investigations tackling the role of the microbiome in the control of blood glucose levels in healthy and T2D conditions, as well as its influence on obesity, may consider including the potential effect that local microbiome metabolites such as SCFAs might have on modulating incretin receptor responses. Overall, the influence of the microbiome on pancreatic islet function and incretin mimetic treatments warrant further investigation.

## Functional interactions of incretin receptors with other GPCRs

**FIGURE 1:**
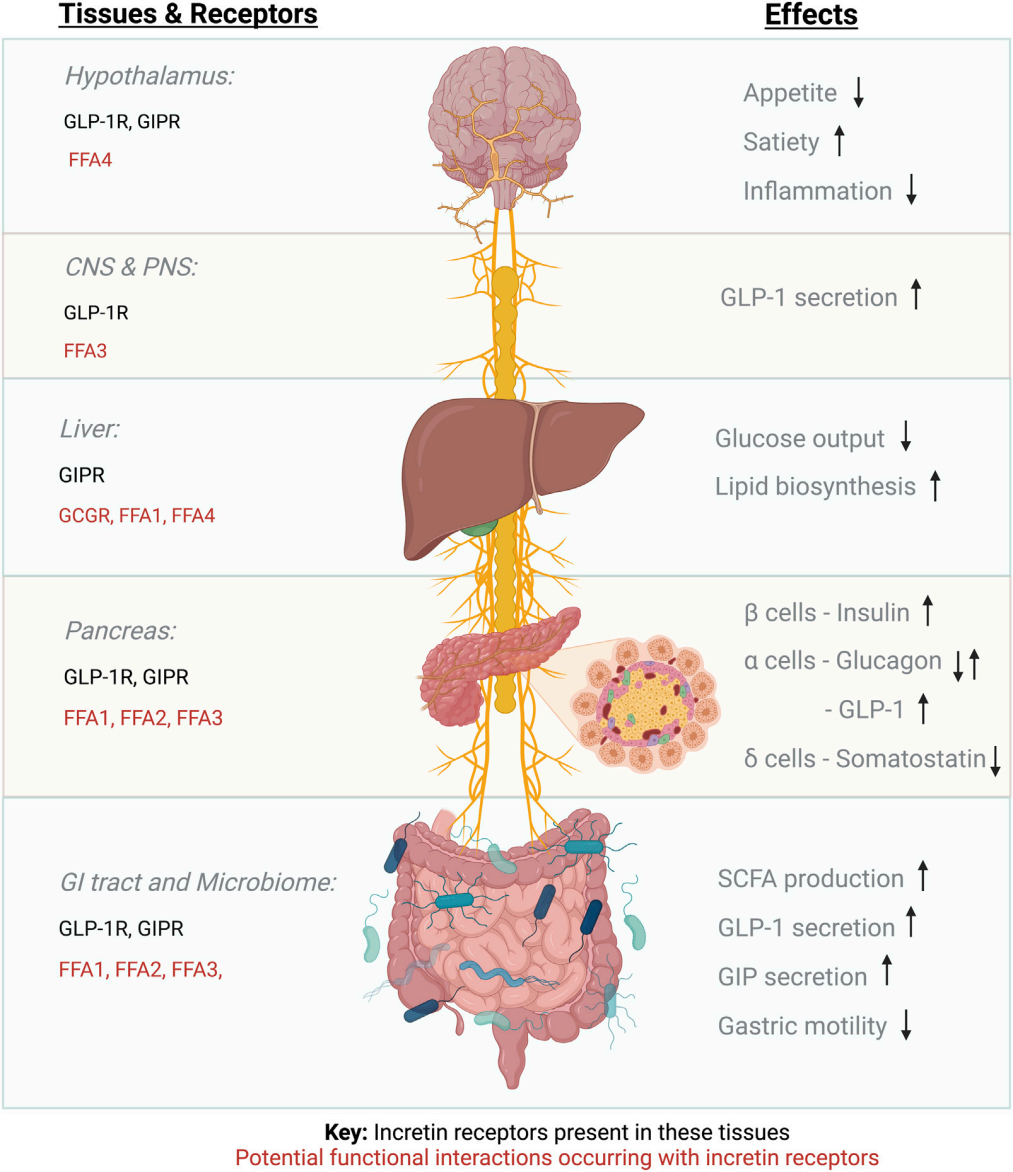
Incretin receptor expression and proposed GPCR functional interactions.

To understand the function of a specific GPCR, it is fundamental to consider the context in which this receptor is signalling, including both physical and functional interactions with other co-expressed receptors (Figure 1). GPCRs can form dimers and oligomers both with themselves and with other GPCRs, suggesting the existence of mechanisms whereby one receptor can influence the function of another, a concept known as receptor crosstalk (Hur and Kim, 2002).

Multiple previous studies have shown that incretin receptors can form heterodimers, as demonstrated, for example, using bioluminescence resonance energy transfer (BRET) (Schelshorn et al., 2012; Whitaker et al., 2012; Dale et al., 2022). In particular (Roed et al., 2015), have shown that both the GIPR and the GCGR form heteromers with the GLP-1R when expressed in HEK-293 cells. This process negatively affects cAMP production and modulates β-arrestin recruitment to the GLP-1R. Another study also carried out in HEK-293 cells showed that incretin heterodimers can form constitutively and that stimulation of GLP-1R with GLP-1 promotes incretin heterodimerisation whereas GIP stimulation reduces it (Al-Zaid et al., 2022). This study additionally showed that GIP stimulation reduces Gα_q_ and β-arrestin recruitment to the GLP-1R whereas GLP-1 stimulation enhances recruitment of Gα_s_ to the GIPR. Future work is required to determine these effects in pancreatic endocrine cells under endogenous levels of receptor expression.

Additionally, there is potential for functional interaction between incretin receptors and other GPCRs, including the SCFA-activated FFARs, to occur in the islets of Langerhans (as previously discussed), but also in the gut and the hypothalamus, all of which could influence incretin therapy responses. Other examples of potential functional interactions might include the receptor for long-chain saturated fatty acids, FFA1, which is also expressed in pancreatic beta cells, where its activation leads to GSIS potentiation *via* Gα_q_ transducer pathways (Kimura et al., 2020), as well as in hepatocytes (Secor et al., 2021) and enteroendocrine L and K cells, where it contributes to incretin hormone secretion, with FFA1 KO mice exhibiting obesity on a low-fat diet (Lu et al., 2021). It is also conceivable that GLP-1R functional interactions might occur in appetite control centres of the hypothalamus: For example, FFA4—the receptor for long-chain polyunsaturated fatty acids, that can regulate energy homeostasis (Dragano et al., 2017), and is expressed alongside the GLP-1R in the arcuate nucleus (Singh et al., 2022). Furthermore, the gut-brain-pancreas axis is connected *via* the parasympathetic vagus nerve with afferent neurons linking the intestinal mucosa, portal vein and hypothalamus. FFA3 is expressed in the enteric sympathetic ganglion (Kimura et al., 2011) and the vagal ganglion (Nøhr et al., 2015) and the GLP-1R is expressed in vagal afferent neurons including the nodose ganglion (Zhang et al., 2020), suggesting the possibility of functional interactions between these two receptors at these neuronal locations. Intraperitoneal injection of butyrate has been shown to regulate food intake in mice *via* the vagus nerve, causing ERK1/2 phosphorylation in nodose ganglion neurons (Goswami et al., 2018); therefore, investigation of potential individual-specific FFAR/incretin receptor functional interactions in the different locations where these receptors are co-expressed is paramount to determine if co-stimulation of both GPCR families might results in enhanced, reduced or biased GLP-1RA responses, an effect which might be regulated by the action of the microbiome.

## Conclusion

In this review, we have discussed several factors that might contribute to an individual’s ability to respond to incretin therapy. Weight loss of at least 5%–10% is known to reduce complications of T2D and improve quality of life (Apovian et al., 2019), and achieving an HbA1c level below 7% (preferably 6.5%) is indicative of sustained blood glucose regulation; therefore, failure to reach these targets under incretin treatment should define a reasonable threshold beyond which a personalised medicine approach needs to be considered. Identifying genetic factors in “non-responders” will help guide treatments, and further research is required to identify muti-targeted approaches that can improve incretin therapies under these conditions. In the meantime, novel dual and tri-incretin agonists are already in development. For example, MEDI0382, a synthetic palmitoylated dual agonist of GLP-1R and GCGR derived from oxyntomodulin, achieved both significant weight loss and blood glucose lowering effects in obese and T2D patients during phase 2 trials (Ambery et al., 2018). G49 is another GLP-1R/GCGR dual agonist shown to improve liver regeneration in non-alcoholic fatty liver disease (Valdecantos et al., 2016). Additionally, a single molecule tri-agonist of GLP-1R, GIPR and GCGR can increase insulin secretion and improve blood glucose handling in mice beyond the cumulative effects of the three individual agonists separately (Khajavi et al., 2018). These benefits were associated with increased Ca^2+^ mobilisation observed in human clonal pancreatic beta cells. Increased Ca^2+^ mobilization may be achieved in future novel treatment strategies by co-targeting Gα_q_-coupled GPCRs present in beta cells, including FFA1 and 2.

The relevance of optimised incretin therapies extends beyond their action on blood glucose regulation and control of body weight, with GLP-1RAs providing beneficial effects for blood pressure disorders (Martins et al., 2020) and neurodegenerative conditions including Alzheimer’s (Bomfim et al., 2012) and Parkinson’s disease (de Pablo-Fernandez et al., 2018). Therefore, with the current strain on health and social care services around the globe, particular investment should be funnelled into this research topic.
